# Vitamin D Status and Risk of Cystic Fibrosis-Related Diabetes: A Retrospective Single Center Cohort Study

**DOI:** 10.3390/nu13114048

**Published:** 2021-11-12

**Authors:** Yiqing Peng, Malinda Wu, Jessica A. Alvarez, Vin Tangpricha

**Affiliations:** 1 Emory College, Emory University, Atlanta, GA 30322, USA; whitney.peng@emory.edu; 2 Division of Endocrinology, Department of Pediatrics, Emory University School of Medicine, Atlanta, GA 30322, USA; malinda.wu@alumni.emory.edu; 3 Division of Endocrinology, Metabolism & Lipids, Department of Medicine, Emory University School of Medicine, Atlanta, GA 30322, USA; jessica.alvarez@emoryed.edu; 4 Atlanta VA Medical Center, Decatur, GA 30300, USA

**Keywords:** cystic fibrosis, vitamin D, vitamin D deficiency, diabetes, epidemiology

## Abstract

Objective: Cystic fibrosis-related diabetes (CFRD) affects up to half of the people with cystic fibrosis (CF) by adulthood. CFRD is primarily caused by pancreatic dysfunction that leads to insufficient insulin release and/or insulin resistance. Exocrine pancreatic insufficiency in people with CF is associated with fat-soluble vitamin malabsorption, including vitamins A, D, E, and K. This study examined the relationship between vitamin D status, assessed by serum 25-hydroxyvitamin D (25(OH)D), and the development of CF-related diabetes (CFRD) in adults with CF. Methods: This was a retrospective cohort study of adults seen at a single CF center. The data were extracted from the electronic medical records and the Emory Clinical Data Warehouse, a data repository of health information from patients seen at Emory Healthcare. We collected age, race, the first recorded serum 25-hydroxyvitamin D (25(OH)D) concentration, body mass index (BMI), and onset of diabetes diagnosis. Log-rank (Mantel–Cox) tests were used to compare the relative risk of CFRD onset in the subjects with stratified vitamin D status and weight status. A sub-group analysis using chi-square tests assessed the independence between vitamin D deficiency and CFRD risk factors, including gender and CF mutation types (homozygous or heterozygous for F508del, or others). Unpaired *t*-tests were also used to compare the BMI values and serum 25(OH)D between the CF adults based on the CFRD development. Results: This study included 253 subjects with a mean age of 27.1 years (±9.0), a mean follow-up time period of 1917.1 (±1394.5) days, and a mean serum 25(OH)D concentration of 31.8 ng/mL (±14.0). The majority (52.6%) of the subjects developed CFRD during the study period. Vitamin D deficiency (defined as 25(OH)D < 20 ng/mL) was present in 25.3% of the subjects. Close to two thirds (64.1%) of the subjects with vitamin D deficiency developed CFRD during the study. Vitamin D deficiency increased the risk of developing CFRD (chi-square, *p =* 0.03) during the course of the study. The time to the onset of CFRD stratified by vitamin D status was also significant (25(OH)D < 20 ng/mL vs. 25(OH)D ≥ 20 ng/mL) (95% CI: 1.2, 2.7, *p* < 0.0078). Conclusion: Our findings support the hypothesis that adults with CF and vitamin D deficiency are at a higher risk of developing CFRD and are at risk for earlier CFRD onset. The maintenance of a serum 25(OH)D concentration above 20 ng/mL may decrease the risk of progression to CFRD.

## 1. Introduction

Cystic fibrosis (CF) is an autosomal recessive disorder caused by a mutation of the cystic fibrosis transmembrane conductance regulator (CFTR) gene. The complications of CF include chronic bacterial infection in the lungs, CF-related diabetes, pancreatic insufficiency, and a decline in lung function [1,2]. Exocrine pancreatic insufficiency leads to fat malabsorption and a deficiency in fat-soluble vitamins, including vitamin A, D, E, and K [3]. Vitamin D deficiency can subsequently lead to CF bone disease and an increased risk of fractures [4,5]. Endocrine pancreatic insufficiency, along with other contributing risk factors, such as body weight, diet, and physical activity, can lead to CF-related diabetes (CFRD). Close to half of the adults with CF will develop CFRD, which is characterized by insufficient insulin production, reduced pancreatic beta cell activity, and insulin resistance [2].

It is well established that vitamin D plays important roles in maintaining bone health, calcium homeostasis in blood, and preventing osteoporosis [6]. Vitamin D insufficiency (25(OH)D < 30 ng/mL), which is commonly observed in people with CF, has been associated with decreased insulin sensitivity and secretion in both animal and human studies [7,8]. The best marker of vitamin D status is the total serum 25-hydroxyvitamin D (25(OH)D), which accounts for both the endogenous production of vitamin D from the skin and dietary intake of vitamin D-containing foods and supplements [9]. A serum 25(OH)D level of less than 30 ng/mL is considered as insufficient, whereas a 25(OH)D of less than 20 ng/mL is considered as vitamin D deficient [10,11]. A recent study suggested that vitamin D status is associated with glucose intolerance and CFRD [12], but it did not address whether vitamin D status influences the risk of CFRD development or time to CFRD onset. The mechanisms by which vitamin D protects against the development of diabetes are uncertain. However, pre-clinical studies have indicated that vitamin D may regulate insulin secretion and improve beta-cell function [13]. 

This current study focuses on the relationship between vitamin D deficiency and CFRD, two common endocrine co-morbidities found in CF. Given the association between vitamin D status and the risk of CFRD, this study aimed to examine the impact of vitamin D status on the onset of CFRD in adults with CF. We hypothesized that low vitamin D status is one of the risk factors for the development of CFRD in adults. The design of the study was a retrospective longitudinal cohort study of adults with CF receiving care at a single center. The data on the subject demographics, serum vitamin D (25(OH)D) measurements, and diabetes status were extracted from the medical records to examine the influence of vitamin D status on CFRD risk. 

## 2. Materials and Methods

### 2.1. Study Design

This was a retrospective chart review examining the relationship between vitamin D status, assessed by serum 25-hydroxyvitamin D (25(OH)D), and development of CFRD. The research study was approved by the Emory Institutional Review Board (IRB). Subjects were adults, age greater than 18, with CF treated by the Emory CF Center and receiving care by the Emory Clinic and Emory Hospital from 2002–2012. Inclusion criteria included a confirmed diagnosis of CF and at least one serum 25(OH)D measurement taken between 1 January 2002 and 31 December 2012. Exclusion criteria included a diagnosis of CFRD at the time of first serum 25(OH)D measurement. Development of CFRD was defined as a diagnosis of CFRD in the medical record, initiation with diabetes medication, fasting glucose ≥ 126 mg/dL, 2hr oral glucose tolerance test (OGTT) glucose ≥ 200 mg/dL, hemoglobin A1C (HgbA1c) ≥ 6.5%, or classical symptoms of diabetes in the presence of a casual glucose ≥ 200 mg/dL. Data from the electronic medical record or the CF Foundation Patient Registry (PortCF) were used to conduct the analysis. We collected data on each subject’s serum 25(OH)D level, body mass index (BMI), date of diabetes diagnosis, presence of pancreatic insufficiency as defined as prescription of pancreatic enzymes, and the number of days since the start of study (1 January 2002) to diagnosis of CFRD, or, if not, to the end of study on 31 December 2012. 

### 2.2. Database and Categorization

Data were obtained from the Emory Clinical Data Warehouse, an electronic database of all clinical laboratory data for the Emory Clinic, Emory University Hospital, and Emory CF Center. Data on pancreatic insufficiency by use of pancreatic enzymes and CFTR mutation were obtained from PortCF. The date of the onset of CFRD was determined by the first date recorded that the subject developed CFRD during the study period. Subjects who did not develop CFRD by the end of the study period were deemed not to have developed CFRD. The first serum 25(OH)D recorded in the electronic medical record during the study period was used to define a subject’s vitamin D status. Vitamin D status deficiency was defined as a 25(OH)D < 20 ng/mL, and insufficient vitamin D level was defined as 25(OH)D < 30 ng/mL. Weight statuses were categorized based on subjects’ BMI. A BMI of more than 25 kg/m^2^ was considered as overweight, and BMI cut-offs greater than 22 kg/m^2^ for female and 23 kg/m^2^ for male subjects were considered as the recommended BMI level for adults with CF [14]. 

### 2.3. Statistical Analysis

Prism 9.0.1 was used to perform statistical analyses. The odds ratios between potential factors contributing to CFRD and vitamin D status were analyzed with 95% confidence interval. Freedom from CFRD was compared using log-rank (Mantel–Cox) tests, and all study subjects were free from CFRD at the beginning of the analysis. Log-rank tests compared the relative risk of CFRD onset in subjects with stratified vitamin D status and weight status. Sub-group analysis using chi-square tests assessed the independence between vitamin D deficiency and CFRD risk factors, including gender and CF mutation types (homozygous for F508del, heterozygous for F508del, or others). Unpaired *t*-test was also used to determine if BMI or first 25(OH)D measures differed between adults with CF who developed CFRD and those that did not.

## 3. Results

### 3.1. Subject Demographics

A total of 267 adults without CFRD were potentially eligible for the study. One patient was excluded due to an implausible BMI value of 43, and 13 subjects were excluded due to missing BMI values. Eventually, 253 subjects met the inclusion criteria, and their baseline demographics are presented in Table 1, stratified by vitamin D deficiency status. During the course of the study, 52.6% of the subjects with CF developed CFRD. During the course of the study, 53.1% of the subjects with insufficient vitamin D (25(OH)D levels < 30 ng/mL) developed CFRD. Using a cut-off value of 25(OH)D < 20 ng/mL, 64.1% of vitamin D deficient subjects (25(OH)D < 20 ng/mL) developed CFRD. 

### 3.2. Time to Onset of Cystic Fibrosis-Related Diabetes

**Figure 1:**
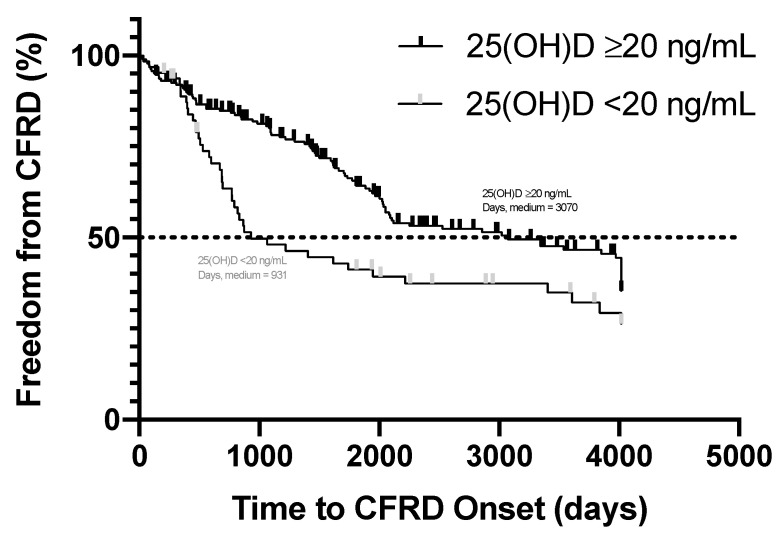
Time in days to cystic fibrosis-related diabetes (CFRD) onset by vitamin D status (25(OH)D < and ≥20 ng/mL). Hazard ratio of the vitamin D deficient subjects to not vitamin D deficient subjects is 1.76 (95% CI: 1.2, 2.7, *p* = 0.0078). The medium for days of CFRD onset was 931 for subjects with vitamin D deficiency and 3070 for those without.

We examined the time to the onset of CFRD by vitamin D status (deficient or insufficient). The time to the onset of CFRD refers to the first instance that a subject had documentation of a CFRD diagnosis as defined in our methods. The log-rank (Mantel–Cox) test was used for the time of CFRD onset by insufficient vitamin D status (25(OH)D < 30 ng/mL) and deficient vitamin D status (25(OH)D < 20 ng/mL). No significant difference was observed in the time to the onset of CFRD in the subjects with CF compared to those with and without insufficient vitamin D status (hazard ratio: 0.84, 95% CI: 0.60, 1.18, *p* = 0.31). However, there was a statistically significant hazard ratio in the time to the onset of CFRD in the subjects with CF compared to those with 25(OH)D < 20 ng/mL versus those with 25(OH)D ≥ 20 ng/mL (Figure 1, hazard ratio: 1.76, 95% CI: 1.2, 2.7, *p* = 0.0078). Based on the survival curve in Figure 1, 50% of the subjects with a deficiency in vitamin D were diagnosed with CFRD at day 931, while, only after 3070 days, 50% of the subjects without deficient vitamin D were diagnosed with CFRD. 

### 3.3. Influence of Other Risk Factors, Including Vitamin D Status, on the Development of CFRD

We evaluated other potential risk factors that may be associated with the development of CFRD. The sub-group analyses using chi-square tests assessed the independence between vitamin D deficiency and CFRD risk factors, including sex, CFTR mutation status (F508del homozygous, F508del heterozygous, or no F508del mutation), and pancreatic enzyme usage. Vitamin D deficiency (25(OH)D < 20 ng/mL) was significantly associated with the development of CFRD (chi square, *p* = 0.03) (Table 2). Insufficient vitamin D status (25(OH)D < 30 ng/mL) was not associated with the risk of CFRD (*p* = 0.87) (Table 2). There were no significant associations between vitamin D deficiency and gender (*p* = 0.10), CF mutation type (*p* = 0.88), or pancreatic enzyme usage (*p* = 0.42). The unpaired *t*-test indicated that the first serum 25(OH)D measure at the start of the study (*p* = 0.83) and average BMI (*p* = 0.08) were not significantly different between the subjects who did and did not develop CFRD (Table 3). 

## 4. Discussion

This retrospective study aimed to investigate the relationship between vitamin D status, assessed by serum 25(OH)D, and the development of CF-related diabetes (CFRD) in a cohort of subjects without CFRD followed over a 10-year period. As expected, we found that more than half of the subjects (52.6%) developed CFRD over the course of the study, which is consistent with previous reports indicating the prevalence of CFRD in the CF population [2]. The results of our statistical analysis demonstrate that subjects with CF had a higher risk of CFRD development, with earlier onset in those with a deficient serum 25(OH)D level at less than 20 ng/mL compared to those with a serum 25(OH)D greater than 20 ng/mL. Our sub-group analysis also indicated that, aside from the susceptibility to CFRD onset, the subjects with and without vitamin D deficiency were not significantly different in other CFRD-related markers, which included gender, BMI, first vitamin D measure, CF mutation types, and pancreatic enzyme usage. 

Our findings support the findings that 25(OH)D > 20 ng/mL may be protective against the development of CFRD. Pincikova et al. found that a serum 25(OH)D less than 20 ng/mL was associated with higher hemoglobin A1c (HbA1c) in a cross-sectional cohort composed of about 900 children and adults with CF living in Scandinavia, suggesting a relationship between vitamin D status and glucose concentrations [12]. The investigators found the association between vitamin D status and HbA1c in children, but such an association was not as strong in adults with CF [12]. In contrast, a similar study performed by Coriati et al. found no relationship between the serum 25(OH)D and measures of glucose status performed by an oral glucose tolerance test in 270 adults with CF in Canada [15].

Studies examining vitamin D status and the risk of diabetes are also mixed in populations without CF. A systematic review by Mitri et al. found that, in eight observational studies, a greater than 500 IU of vitamin D intake per day was associated with a 13% decreased risk of type II diabetes compared to a vitamin D intake of less than 200 IU per day [16]. However, there were no associations found in the development of type II diabetes in the post hoc analyses of randomized controlled trials [16]. A meta-analysis of vitamin D and the risk of type I diabetes found, in four case control studies, that vitamin D supplementation in early childhood reduced the risk of type I diabetes compared to children not supplemented with vitamin D [17]. A recent large multi-center randomized controlled trial found that vitamin D supplementation did not protect against the progression from prediabetes to type II diabetes in adults [18]. Therefore, the current literature is mixed in regard to the potential effect of vitamin D on the development of diabetes, with some potential protection of vitamin D in children with type I diabetes but no benefits seen in adults with type II diabetes and prediabetes.

Vitamin D is thought to be protective against the development of type I diabetes by modulating the immune system by dampening the autoimmune response, decreasing self-destructive islet cell auto-antibodies produced by beta-cells, and, thus, preserving the beta-cell mass of the pancreas. In murine models of type I diabetes mellitus, supplementation with the active form of vitamin D, calcitriol, prevented the onset of diabetes, which further supported the role of vitamin D in the prevention of type I diabetes [19,20]. For type II diabetes, vitamin D was thought to enhance insulin sensitivity; however, a recent meta-analysis of 18 randomized controlled trials found no benefit of vitamin D supplementation on the markers of insulin sensitivity [21]. Finally, a recent meta-analysis of vitamin D on the markers of inflammation and oxidative stress in people with diabetes found that vitamin D reduced C-reactive protein and malondialdehyde levels, both markers of inflammation. The supplementation of vitamin D also increased the nitric oxide release, serum antioxidant capacity, and total glutathione concentrations, favorable changes in oxidative stress [22]. Since CFRD shares some features of type I and II DM, vitamin D may have many roles in the prevention of diabetes by reducing inflammation, oxidative stress, and, potentially, by preserving beta-cell mass.

Optimal vitamin D status has been associated with the decreased risk of diabetes and prediabetes in populations without CF [23]. In addition, better vitamin D status among patients with diabetes and prediabetes is associated with a reduced risk for all-cause mortality [24,25]. However, in a randomized controlled trial of subjects with prediabetes and without CF, intervention with 4000 IU of vitamin D for 2.5 years did not reduce the progression to diabetes compared to a placebo [18]. Despite the protective mechanisms of vitamin D, as outlined above, and the strong association between vitamin D status and the risk of diabetes, clinical trials have been negative in establishing a role of vitamin D supplementation for prevention of progression to diabetes. Thus, vitamin D intervention may need to be provided much earlier in life before beta cell dysfunction and/or vitamin D deficiency represents poor general health that is not modifiable by supplementation with vitamin D.

Our study found that a serum 25(OH)D > 20 ng/mL was protective against the risk of CFRD. However, larger studies in the general population using the NHANES data have suggested that a cut-off of 30 ng/mL was protective against diabetes and metabolic syndrome [26]. The CF Foundation recommends a 25(OH)D level of greater than 30 ng/mL to protect against CF bone disease [27]. The 25(OH)D cut-off of 30 ng/mL to establish vitamin D sufficiency is based on recommendations in people without CF, including a study showing no osteomalacia found on bone biopsy in adults with 25(OH)D concentrations greater than 30 ng/mL [28]. Some investigators have proposed even higher concentrations of 25(OH)D to prevent elevations in PTH concentrations to prevent bone loss [29]. While there is a general consensus that a 25(OH)D concentration of above 30 ng/mL is optimal for bone health in CF, it is not known if different thresholds are protective against conditions such as CFRD, pulmonary exacerbations, or infections. Furthermore, different organ systems may need different thresholds for optimal health. Finally, the duration that 25(OH)D concentrations need to be sufficient is not known either. Studies indicate that the short-term correction of vitamin D status in CF, such as at the time of pulmonary exacerbation, does not change the course of the disease [30]. Until data are available to determine the optimal 25(OH)D concentration for all the health conditions in CF, clinicians should still address vitamin D status by supplementation or encouraging outdoor sunlight exposure in people with CF [9,31].

This is the first study that analyzed the long-term impact of vitamin D status on the onset of diabetes specifically in adults with CF. Limitations to our study include the limited reports on the use of any pancreatic enzymes, the interaction of vitamin D and CFTR modulator therapy, and the types of CFTR mutations in the entire subject population. Another limitation is the lack of data on other potential risk factors for the development of CFRD. Since this was a retrospective study, we lacked information on diet, physical activity, medications, and other health conditions. Only 68 of the 253 subjects had records of CFTR mutation and whether they were prescribed with pancreatic enzymes during their treatment regimes. Although CF mutation types and pancreatic enzyme usage were used as indicators for pancreatic insufficiency, we were unable to draw substantial conclusion due to the sample size of less than 20 when stratified based on vitamin D status. Understanding the CFTR mutation type and the usage of pancreatic enzyme is important to adjust for equal sampling distribution because both factors can potentially affect vitamin D deficiency and diabetes onset. Having an F508 del homozygous mutation is associated with more severe CF complications and a higher risk of pancreatic defects [1]. Therefore, adults with an F508del homozygous mutation might have a higher risk of diabetes development despite the vitamin D status.

Our findings support the hypothesis that adults with CF and vitamin D deficiency are at a higher risk of developing CFRD and are at risk for earlier CFRD onset. We found that a serum 25(OH)D concentration above 20 ng/mL may decrease the risk of the progression to CFRD. However, what is not clear and cannot be answered in this retrospective study is whether providing vitamin D supplements will actually decrease the progression to CFRD. Future randomized prospective studies should evaluate whether vitamin D supplementation with vitamin D in adults and/or children can decrease the progression to CFRD.

## Figures and Tables

**Table 1:** Baseline subject demographics stratified by vitamin D deficiency at 20 ng/mL.

	All Subjects	Vitamin D Deficient (25(OH)D < 20 ng/mL)	Vitamin D Not Deficient (25(OH)D ≥ 20 ng/mL)
Subjects n (%)	253	64 (25.3)	189 (74.7)
Age at Entry, y	27.1 (±9.0)	26.9 (±8.3)	27.1 (±9.2)
Gender, male	132 (52.2)	39 (60.1)	93 (49.2)
Gender, female	121 (47.8)	25 (39.1)	96 (50.8)
Race, Caucasian or White ^a^	231 (91.3)	47 (73.4)	186 (97.4)
Race, African American or Black ^a^	18 (7.1)	16 (25)	2 (1.1)
Days without Diabetes Mellitus ^b^	1917.1 (±1394.5)	2161.2 (±1627.6)	2410.7 (±1667.6)
BMI, kg/m^2^	21.8 (±3.4)	21.8 (±3.8)	21.7 (±3.3)
BMI at goal ^c^	165 (65.2)	45 (70.3)	120 (63.5)
BMI ^d^, <25 kg/m^2^	211 (83.4)	53 (82.8)	158 (83.6)
Developed CFRD ^e^	133 (52.6)	41 (64.1)	92 (48.7)
25(OH)D, ng/mL	31.8 (±14.0)	12.5 (±4.4)	36.9 (±15.5)

Entries are means (±standard deviation), n (%), or % (n). ^a^ Six missing values of race not reported. ^b^ Days, since study start on 1 January 2002. End date was the day when the subject was diagnosed with diabetes, or, if the subject never developed diabetes, 31 December 2012. ^c^ The BMI goal for adults with CF is greater than 22 kg/m^2^ for females and 23 kg/m^2^ for males. ^d^ BMI at or above 25 kg/m^2^ is considered overweight. ^e^ Developed diabetes between 2002–2012.

**Table 2:** Sub-group analysis of vitamin D deficiency/insufficiency with selected demographic factors.

	Compared Value	X^2^. Df	*p*-Value
Vitamin D Deficiency ^a^	CF Mutation Type ^b^	0.27, 2	0.88
Vitamin D Deficiency	Develop CFRD	4.54, 1	0.03 *
Vitamin D Deficiency	Gender, Male or Female	2.63, 1	0.10
Vitamin D Deficiency	Pancreatic Enzyme Usage ^c^	0.66, 1	0.42
Develop CFRD ^d^	Gender, Male or Female	0.43, 1	0.51
Vitamin D Insufficiency ^e^	Develop CFRD	0.03, 1	0.87

Results from sub-group analysis based on chi-square test analysis. ^a.^ Subjects stratified by vitamin D deficiency at 25(OH)D < 20 ng/mL. ^b.^ CF mutation types categorized by F508del homozygous, F508del heterozygous, or no F508del mutation ^c.^ Pancreatic enzyme usage, yes or no, recorded at the start of study ^d.^ Subjects stratified by whether they were diagnosed with CFRD ^e.^ Subjects stratified by vitamin D insufficiency at 25(OH)D < 30 ng/mL. An asterisk (*) indicates statistical significance at *p* < 0.05.

**Table 3:** Sub-group analysis of subjects developing CFRD during the course of the study.

	Compared Value	t-Score, df	*p*-Value
Develop CFRD ^a^	First 25(OH)D, ng/mL ^b^	0.22, 251	0.83
Develop CFRD	BMI, kg/m^2^	1.74, 251	0.08

Results from sub-group analysis based on unpaired *t*-test. ^a^ Subjects stratified by whether they were diagnosed with CFRD ^b.^ First serum vitamin D measure obtained from subjects at the start of the study.
